# Comparative Genomic Analysis Reveals 2-Oxoacid Dehydrogenase Complex Lipoylation Correlation with Aerobiosis in Archaea

**DOI:** 10.1371/journal.pone.0087063

**Published:** 2014-01-28

**Authors:** Kirill Borziak, Mareike G. Posner, Abhishek Upadhyay, Michael J. Danson, Stefan Bagby, Steve Dorus

**Affiliations:** 1 Department of Biology, Syracuse University, Syracuse, New York, United States of America; 2 Department of Biology & Biochemistry, University of Bath, Claverton Down, United Kingdom; 3 Centre for Extremophile Research, University of Bath, Claverton Down, United Kingdom; Belgian Nuclear Research Centre SCK/CEN, Belgium

## Abstract

Metagenomic analyses have advanced our understanding of ecological microbial diversity, but to what extent can metagenomic data be used to predict the metabolic capacity of difficult-to-study organisms and their abiotic environmental interactions? We tackle this question, using a comparative genomic approach, by considering the molecular basis of aerobiosis within archaea. Lipoylation, the covalent attachment of lipoic acid to 2-oxoacid dehydrogenase multienzyme complexes (OADHCs), is essential for metabolism in aerobic bacteria and eukarya. Lipoylation is catalysed either by lipoate protein ligase (LplA), which in archaea is typically encoded by two genes (*LplA-N* and *LplA-C*), or by a lipoyl(octanoyl) transferase (LipB or LipM) plus a lipoic acid synthetase (LipA). Does the genomic presence of lipoylation and OADHC genes across archaea from diverse habitats correlate with aerobiosis? First, analyses of 11,826 biotin protein ligase (BPL)-LplA-LipB transferase family members and 147 archaeal genomes identified 85 species with lipoylation capabilities and provided support for multiple ancestral acquisitions of lipoylation pathways during archaeal evolution. Second, with the exception of the Sulfolobales order, the majority of species possessing lipoylation systems exclusively retain *LplA*, or either *LipB* or *LipM*, consistent with archaeal genome streamlining. Third, obligate anaerobic archaea display widespread loss of lipoylation and *OADHC* genes. Conversely, a high level of correspondence is observed between aerobiosis and the presence of *LplA/LipB/LipM*, *LipA* and OADHC *E2*, consistent with the role of lipoylation in aerobic metabolism. This correspondence between OADHC lipoylation capacity and aerobiosis indicates that genomic pathway profiling in archaea is informative and that well characterized pathways may be predictive in relation to abiotic conditions in difficult-to-study extremophiles. Given the highly variable retention of gene repertoires across the archaea, the extension of comparative genomic pathway profiling to broader metabolic and homeostasis networks should be useful in revealing characteristics from metagenomic datasets related to adaptations to diverse environments.

## Introduction

Culture-independent, metagenomic analyses have been particularly successful in advancing our knowledge of microbial abundance across diverse ecological niches (reviewed by [1]). Nonetheless, few studies have leveraged the wealth of genomic data across diverse archaeal taxa to explore adaptation to extreme archaeal environments although this must have a functional basis in genomic diversification [2], [3], [4], [5]. Recent experimental studies have begun to utilize metagenomic data to decipher evolutionary processes [6] but substantial obstacles remain in applying such approaches to the complex biotic and abiotic interactions of natural populations (reviewed by [7]). To what extent can comparative genomic approaches inform our understanding of the evolution and functional capacity of organisms that cannot be cultured or studied in the laboratory? Further, can abiotic characteristics of extremophile habitats be inferred directly from the analysis of metagenomic data?

Archaeal evolution has been dominated by reductions in genome complexity and the retention of highly variable genetic architectures across lineages ( [8] and reviewed by [9]). Recent analyses reveal two distinct phases of archaeal genome evolution. The first, the innovation phase, is associated with an increase in genome complexity and an associated increase in gene families to an average of approximately 2500 gene families. The second, the reductive phase, is characterized by genome streamlining and the retention of a more minimal, and potentially heterogeneous, gene repertoire (1400–1800 gene families) [10]. This persistent genomic streamlining has radically altered the repertoires of even the most highly conserved gene classes, including those involved in translation, replication, cell division and DNA repair, and is thus central to functional diversity across the domain [11]. In addition to the diversifying impact of differential gene loss across taxa, archaeal genome analyses have revealed notable exceptions where horizontal gene transfer (HGT) has been a prevalent force. For example, gene flow from eubacteria to Halobacteriales has contributed to the absence of reductive genome evolution in this archaeal order [10]. We therefore propose that gene repertoire heterogeneity, particularly associated with metabolism and homeostasis, may reflect archaeal adaptation to, and exploitation of, a remarkable diversity of environments. We assess this possibility by considering aerobiosis within archaea because (i) archaea display tremendous diversity in their utilization and tolerance of aerobic environments and (ii) aerobiosis pathways have been well characterized biochemically. Lipoylation, the covalent attachment of lipoic acid to the dihydrolipoyl acyltransferase (E2) subunit of 2-oxoacid dehydrogenase multienzyme complexes (OADHCs), is essential for metabolism in aerobic bacteria and eukarya (reviewed by [12], [13]). Specifically, OADHC lipoylation is required for channeling substrates between the active sites of the three protein subunits of OADHCs: 2-oxoacid decarboxylase (E1), E2 and dihydrolipoamide dehydrogenase (E3). The lipoyl domain of E2 (E2lipD) is the post-translational modification target. The mechanisms of lipoylation have been studied to varying extents in all domains of life [14], [15], [16], [17]. In *Escherichia coli*, lipoylation is catalyzed by two routes: lipoic acid synthetase (LipA) and lipoyl(octanoyl) transferase (LipB), or lipoate protein ligase (LplA) [18]. LipB and LipA work in tandem: LipB catalyzes the covalent attachment of octanoic acid to the E2 lipoyl domain, and then LipA introduces sulphur atoms at the C6 and C8 positions. Alternatively, LplA can catalyse both conversion of lipoic acid to lipoyl-AMP and subsequent covalent attachment of the lipoyl moiety to E2lipD [19], [20]. It is noteworthy that greater diversity in lipoyl biosynthesis has been observed in other eubacteria, including an alternative octanoyl transferease, LipM, and a lipoyl-scavenging protein, LipL, in Firmicutes [17], [21], [22]. In eukaryotes and most bacteria, LplA is encoded by a single gene, whereas studies in the archaeon *Thermoplasma acidophilum* revealed distinct genes, *LplA-N* and *LplA-C*, encoding proteins that correspond to the N- and C-terminal domains of *E. coli* LplA and that are both required for E2 lipoylation [23], [24], [25]. The distribution and genomic characteristics of lipoylation systems have yet to be studied across archaea.

Based on their well characterized biochemical interaction, we propose that genomic retention of the components of the OADHC lipoylation pathway, including lipoylation enzymes and E2, may serve as a diagnostic marker for aerobic metabolism. We have therefore examined their evolutionary retention across available archaeal genomes in the context of the following predictions. First, co-retention of *LplA*, *LipB* or *LipM* is unexpected given the widespread genomic streamlining observed in archaea. Second, the octanoyl transferases, LipB and LipM, would appear to be unlikely to be the pervasive archaeal lipoylation system given their enzymatic preference for octanoic acid, a product of fatty acid (FA) biosynthesis. FA biosynthesis was believed to be completely absent from archaea [26], although archaeal FA synthase pathways have recently been identified [27]. Although the prevalence of archaea FA biosynthesis has yet to be carefully examined, we suggest that the genomic presence of octanoyl transferases may be a reliable indicator of this biochemical capacity. Third, evolutionary loss of lipoylation, including lipoylation enzymes and their E2 substrates, may be widespread in anaerobic archaea, particularly those that are obligate anaerobes or display poor oxygen tolerance. Targeting this well characterized metabolic pathway also provides a general assessment of the robustness of genomic inferences about the metabolic regimes of difficult-to-study microbes whose genomes are highly represented in environmental metagenomic studies [28], [29], [30].

## Materials and Methods

### Lipoylation System Classification

Lipoylation systems across the three domains of life were surveyed to assess the presence of each lipoylation system amongst archaea. To do so we characterized the genomic composition of lipoylation systems and OADHC lipoic acid acceptor protein (E2) in 147 archaeal species, including 43 Crenarchaeota, 96 Euryarchaeota, 5 Thaumarchaeota, 1 Korarchaeum, 1 Nanoarchaeum and 1 Aigarchaeum of which 20 are genome sequences from metagenomic environmental samples. First, an analysis of all 11,826 protein domains within the Pfam BPL_LplA_LipB cofactor transferase family protein domain (PF03099) [31] was conducted. Domain protein sequences were aligned using the MAFFT iterative refinement method [32], and a neighbor-joining phylogenetic tree was constructed with the NINJA algorithm, using the default parameters [33]. The resultant phylogeny resolved clades that corresponded to LplA, LipM, LipL and LipB based on existing biochemical characterization for proteins within each clade [16], [17], [19], [34], [35], [36] This Pfam analysis thus provided a preliminary catalogue of archaeal lipoylation.

### Comparative Genomic Analysis

To address the possible incomplete annotation of archaeal lipoylation proteins in the Pfam PF03099 database, homology-based approaches were used to confirm and expand the identification of LplA, LipM and LipB in the 147 archaeal genomes (Table S1). *T. acidophilum* LplA-N (Q9HKT1), *F. acidarmanus* LipB (S0AQU0) and *M. arvoryzae* LipM (Q0W155) protein sequences were obtained from UniProt and searched against annotated archaeal protein databases (NCBI Microbial Genomes) using BLASTp (E-threshold = 1E-10) to identify a representative sequence with the highest homology in each of the thirteen taxonomical groups analyzed. These “best-hit” representative sequences were then searched against available genome sequences using tBLASTn within their respective taxonomical group to determine the presence and copy number of each gene. In species where no homologous genes were identified, PSI-BLAST (E-threshold = 0.001; 2 iterations maximum) was also used to confirm the absence of any related sequence. Both BLASTp and tBLASTn results were manually assessed to ensure identification of lipoylation proteins and exclusion of biotinylation proteins, based on the Pfam phylogenetic classification.

A similar approach was used to assess the presence of the lipoic acid adenylation domain LplA-C (using *T. acidophilum* Q9HKT2), the octanoyl synthase LipA (using *F. picrophilus* S0AQU0), the eubacterial octanoyl transferase LipL (using *B. subtilis* P54511), and lipoylation substrates, including the dihidrolipoyl transferase (E2) subunit of the OADHC (using *T. acidophilum* Q9HIA5). The *B. subtilis* LipL sequence was used because no annotated archaeal LipL exists. BLASTp and tBLASTn were conducted on these sequences as described above. Again, manual curation was employed to exclude proteins with the LplA-C domain that exist as part of lipoylation and biotinylation proteins, non-LipA radical SAM proteins, and biotinylation targets. In order to identify lipoylation targets exhaustively, the lipoyl domain of *T. acidophilum* E2 was used as a PSI-BLAST query. Using an E-value cutoff of 0.001, PSI-BLAST was iterated until convergence (four iterations). Due to the abundance of biotinyl domains in the results, maximum likelihood phylogenetic analyses were employed to differentiate between the two targets (see below). The lipoyl domains were also differentiated from biotinyl domains based on protein domain architecture and sequence annotation. The resultant lipoyl domain-containing proteins were classified based on their domain architectures, revealing three distinct classes: true dihydrolipoyl transferase proteins (based on the presence of the acyltransferase catalytic domain, PF00198), glycine cleavage protein H (GcvH) (based on annotation and high homology with biochemically characterized bacterial GcvH), and single domain proteins (containing only the lipoyl domain). Confirming our Pfam results, no archaeal LipL proteins were identified in either BLASTp or tBLASTn analyses.

Additionally, sequence motif analysis was conducted as a validation step. To confirm LplA-N identification we examined conservation amongst two essential motifs to confirm LplA-N sequences (Fig. S1). The first is RRXTGGG(G/A/S/T)(A/I/V)(I/F/Y)HD with the second R and first two Gs forming the core. In the *T. acidophilum* LplA-N:LplA-C complex structure [25], this motif lies at the functional interface of LplA-N with LplA-C and includes the lipoate binding loop. The second conserved motif is G(R/K)K(I/L/V)SGX(A/G)Q, with occasional substitution of the first G, the S and Q. This motif, corresponding to residues 143–151 of *T. acidophilum* LplA-N, forms part of β9 in *T. acidophilum* LplA-N:LplA-C. β9 is located adjacent to the lipoate binding loop [25] and in lipoyl-AMP-bound structures the conserved K and G at the third and sixth positions respectively of the motif are involved in interactions with the adenine and lipoyl parts of lipoyl-AMP [37].

### Maximum Likelihood Phylogenetic Analysis

The phylogenetic relationships between LplA, LipM and LipB in archaea, eubacteria and eukaryotes, as well as all archaeal proteins containing biotinyl and lipoyl domains were analyzed using a maximum likelihood phylogenetic approach. Protein sequences were retrieved from UniProt for all archaea identified in the previous analyses, major eukaryotic species (*S.cerevisiae, D. melanogaster*, *M. musculus* and *H. sapiens*) and eubacteria representing Actinobacteria (*S. coelicolor),* Bacteroidetes (*B. thetaiotaomicron*), Firmicutes (*B. subtilis* and *S. aureus*) and Proteobacteria (*E. coli* and *B. pseudomallei).* Multiple sequence alignment was conducted using the L-INS-i algorithm of MAFFT [32]. Bootstrapped maximum likelihood phylogenetic analyses were done with empirical amino acid frequencies, sub-tree pruning and regrafting topology search, and a parsimony starting tree using the PhyML package [38].

### Horizontal Gene Transfer (HGT) Analysis

The codon based approach of Davis and Olsen (2009) was used to calculate modal codon usage for the 147 archaeal species surveyed above and to detect significant codon usage outliers as putative HGT events [39]. Protein coding sequences were downloaded for all species and genes were deemed as recent horizontal acquisition events if the codon usage was significantly different from the whole genome modal frequency using a threshold of p<0.10, as suggested by Davis and Olsen (2009), and a more conservative threshold (p<0.05). Statistical comparison of the frequency of HGT between gene sets was conducted using a two-tailed Chi-square test with Yates correction.

### Archaeal Metabolic Environments

The categorization of archaeal metabolic environments, particularly relating to aerobiosis and oxygen tolerance, was based on a detailed curation of available literature and the Genomes Online Database (GOLD v4.0; [40]). The availability of phenotypic and habitat information is highly variable amongst archaea, particularly given the expanded use of metagenomic environmental sampling. In some archaeal orders, relevant data were limited to a subset of the member species.

## Results

### Archael Lipoylation Pathway Heterogeneity

**Figure 1:**
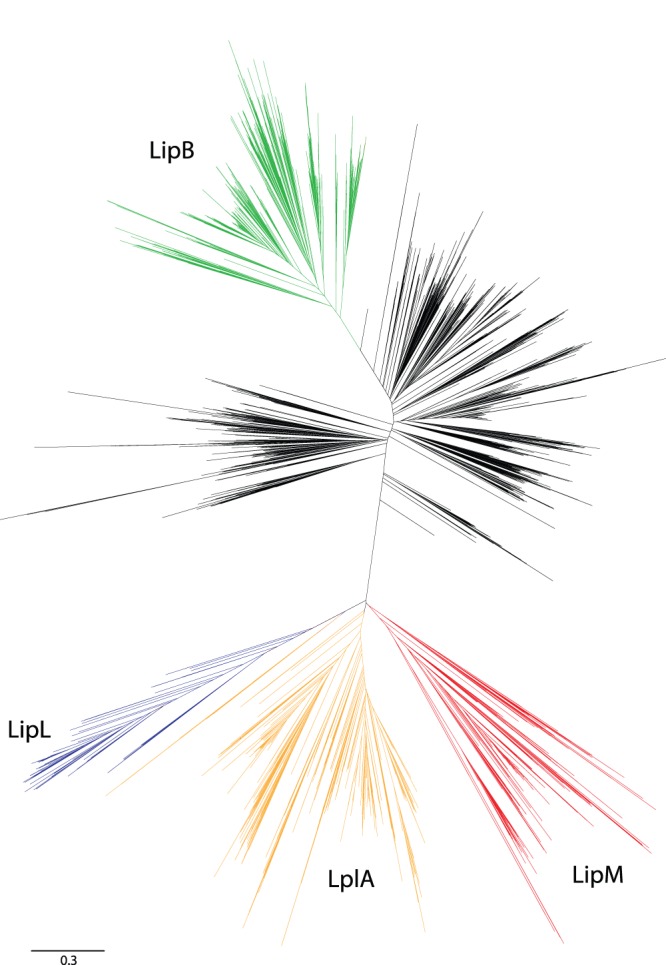
Phylogenetic analysis of the biotin-lipoate A/B protein ligase family. Neighbor-joining phylogenetic tree of the cofactor transferase domain (Pfam03099) that includes 11,826 biotin and lipoate-ligase proteins and octanoyl-carrier proteins from eukaryotes, eubacteria and archaea. Annotation of the five broad protein domain clades as LipM (red), LplA (orange), LipL (blue), LipB (green) and biotin protein-ligase (black) clades was based upon the presence of biochemically characterized proteins within each protein set. In total, 295 archaeal sequences were included in this analysis with 161 residing in the biotin-ligase clade (54.5%) and the remainder residing in the LipM, LplA or LipB clades.

To broadly characterize the distribution of lipoylation pathways across archaea we conducted a comprehensive analysis of proteins within the biotin and lipoate B/A ligase and octanoyl carrier domain family (Pfam03099). Neighbor-joining tree construction for all domain sequences within this family (n = 11,826) resulted in five broad clades, encompassing the biotin ligase, LplA, LipB, LipL and LipM protein groups (Fig. 1). Archaeal representatives were identified amongst all these clades with the exception of LipL, which has been previously identified only in bacterial Firmicutes [17]. In total, 126 archaeal lipoylation proteins were identified and formed the basis of our subsequent assessment of the prevalence of lipoylation proteins across 147 archaeal species. These searches revealed 16 additional archaeal proteins in 14 taxa, resulting in a total of 142 lipoylation proteins in 85 species (Table S1).

**Figure 2:**
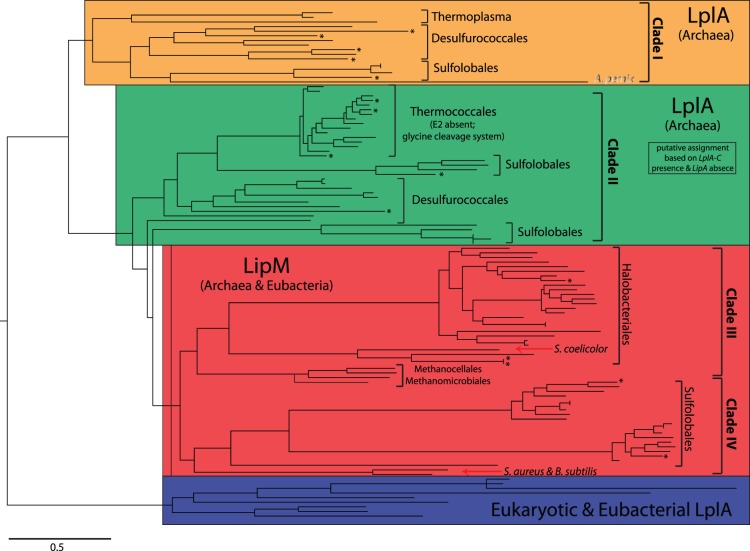
Phylogenetic analysis of LipM and LplA. Maximum likelihood phylogenetic tree including 131 LipM and LplA sequences from archaea, LplA sequences from major eukaryotic species (*S. cerevisiae, D. melanogaster*, *M. musculus* and *H. sapiens*) and LplA and LipM sequences from eubacteria representing Actinobacteria (*S. coelicolor),* Bacteroidetes (*B. thetaiotaomicron*), Firmicutes (*B. subtilis* and *S. aureus*) and Proteobacteria (*E. coli* and *B. pseudomallei).* Putative cases of horizontal gene transfer are indicated (asterisk) and major phylogenetic clades are highlighted: archaeal LplA (Clade I - orange; Clade II - green), LipM (red), and eukaryotic and eubacterial LplA (blue). The full phylogenetic tree including species names and bootstrap values is provided Figure S2.

The LipB lipoylation system was found to be the least prevalent (11 genes within 9 species) and was straightforward to distinguish given the substantial sequence divergence between LipB and LipM/LplA proteins (Fig. 1). It is noteworthy that all species possessing LipB also possess the lipoate synthase LipA and E2 and thus have a complete E2 lipoylation pathway. Due to the higher levels of homology, a full maximum likelihood phylogenetic analysis was conducted to distinguish between LipM and LplA sequences. Of the 131 protein sequences analyzed, 63 genes in 44 taxa were identified within two closely related monophyletic clades that were associated with Actinobacterial and Firmicute LipM, respectively (Fig. 2; Clade III and IV). The remaining 68 sequences in 50 taxa consist of putative LplA-N proteins. Amongst these, the proteins found in Clade I are LplA based upon (i) their phylogenetic relationship with the biochemically characterized *T. acidophilum* LplA, (ii) manual inspection of diagnostic amino acid residues within the catalytic domain (see Methods) and (iii) the genomic presence of the other part of this bipartite system, LplA-C. Despite the closer phylogenetic relationship of Clade II to LipM, we propose that proteins in this clade are LplA-N, and not octanoyl transferases, based on the correlated presence of LplA-C and the absence of LipA in the genomes of these taxa (Table S1); however this classification should be considered provisional in the absence of additional biochemical data. Based on the fact that the octanoic acid transferred to E2 by LipM requires LipA for conversion to a lipoyl group, the genomic presence of LipA was used to independently confirm LipM in Clades III and IV. As expected, LipA was identified in 38 of the 44 genomes that possess LipM, thus confirming the complete lipoylation pathway in those taxa. Additionally, LipA was absent from all species with LplA as a sole transferase system, with the exception of *A. pernix*. Thus, the combination of complementary phylogenetic and genomic approaches provides a substantive basis for differentiating between LipM and LplA.

### Origins of Archaeal Lipoylation Pathways

Our phylogenetic analysis provides support for ancestral monophyletic origins of lipoylation systems prior to the divergence of species within archaeal orders. A pattern consistent with this is observed in several clades, including LplA in Thermococcale, LipM in Methanocellales, Halobacteriales and Sulfolobales (Fig. 2), and LipB in Thermoproteales and the closely related Aigarchaeota (represented by *C. subterraneum*) (Fig. S3). In several cases it is also possible to infer the likely source of lipoylation system acquisition. For example, the inclusion of *Streptomyces* within LipM Clade III, which includes diverse Halobacteriales and Methanocella, is consistent with acquisition from an ancestral Actinobacterial species. Similarly the presence of *Staphylococcus* and *Bacillus* within Clade IV is indicative of an independent ancestral gene transfer of LipM from an ancestral Firmicute. Lastly, in Thermoproteales and Aigarchaeota, LipB displays a monophyletic relationship with *E. coli* and *Burkholderia* (Proteobacteria) LipB (Fig. S3). These observations are most parsimoniously explained by ancestral acquisition events although it is difficult to exclude the possible effects of historical HGT amongst archaeal taxa on the contemporary phylogenetic distribution of these systems. The evolutionary origins of the LplA system in archaea are more difficult to reconstruct as archaeal LplA sequences are very distantly related to LplA in eubacteria and eukaryotes. However, this observation in conjunction with the deep evolutionary branches across numerous LplA clades is most consistent with ancient origins of archaeal LplA systems and potential loss during archaeal evolution.

To explicitly examine the prevalence of HGT in the evolution of archaeal lipoylation systems, we performed codon usage bias analysis of all 147 archaeal genomes in our dataset. The modal codon usage method revealed that 14.1% (20 of 142; p<0.10) of lipoylation genes show significantly different codon usage from the genomic mode, consistent with horizontal gene transfer events (Table S1). The frequency of significant HGT events involving lipoylation genes is significantly lower than the observed genome average (34.5% across 147 genomes; p<0.0001) and recently published estimates [41]. Although putative HGT events were distributed across many archaeal orders (Fig. 2), these putative events were significantly concentrated amongst the 20 species possessing multiple copies of lipoylation genes in their genomes (χ^2^ = 3.84. p = 0.025). Amongst the 20 putative HGT events, 13 also occur amongst closely related taxa as evidenced by their phylogenetic proximity (Fig. 2). For example, three HGT events involving LipB were found to be concentrated in Thermoproteales (*Pyrobaculum aerophilum* str. IM2, *Pyrobaculum calidifontis* JCM 11548, and *Pyrobaculum oguniense* TE7) and these genes are closely related to all other Thermoproteales LipB genes (Fig. S3). A second possible hallmark of HGT events between closely related taxa would be the acquisition of a second copy of the same lipoylation gene from a closely related sister taxon. This was observed in 9 out of 10 events in species with multiple lipoylation genes (Table S2). Taken together, HGT is not particularly prevalent amongst lipoylation genes (in comparison to the genome average) and the enrichment of recent HGT events in taxa with multiple genes is more consistent with transient increases in copy number (and the potential establishment of functional redundancy), which are subsequently returned to a single-gene state by gene loss.

### Lipoylation Pathway Retention in Archaea

Genomic streamlining in archaea has been well documented [8], [9] and may extend to ancestrally acquired lipoylation pathways in archaea. Consistent with this prediction, the majority of archaea capable of lipoylation (79%, 67 of 85 species) exclusively retain only one transferase system: either *LplA* or *LipM* or *LipB* (Table S2). Sulfolobale species, of which 8 out of 16 are isolates of *S. islandicus*, are the primary exception, possessing multiple copies of both *LplA* and *LipM*. As mentioned previously, only 9 species (excluding Sulfolobales) retain multiple lipoyl transferase genes. None of these cases includes the retention of multiple distinct lipoylation transferase systems (for example, *LipM* and *LplA* or *LipM* and *LipB*) and 6 of these display evidence of being the result of a recent HGT event. These observations, in conjunction with our phylogenetic analyses, are consistent with ancestral lipoylation system acquisition events in archaea. Furthermore, the marginally greater retention of *LipM* and *LipB* (53 species) relative to *LplA* (50 species) is also noteworthy as it was previously thought that FA biosynthesis, the source of octanoic acid, was absent (or taxonomically restricted) in archaea. Contrary to this view, the widespread identification of octanoyl transferases provides strong complementary support for FA biosynthesis across diverse archaea [27].

### Correspondence between Lipoylation Pathways and OADHC Substrate E2

Addition of a lipoyl moiety to the E2 subunit of OADHCs is essential for aerobic metabolism. We therefore catalogued the presence of *E2* across archaeal genomes and, as expected, our results show a widespread correlation between the presence of *E2* and lipoylation systems. Specifically, all species (9 out of 9) possessing *LipB* have an intact lipoylation pathway, also possessing lipoyl synthetase *LipA* and *E2*. Similarly, 82% of species (36 of 44) possessing *LipM* also possess *LipA* and *E2*. Exceptions to this include 7 Sulfolobales (*A. hospitalis* W1, all three Metallosphaera species, *S. acidocaldarius* DSM 639, *S. islandicus* REY15A, and *S. tokodaii* str. 7) and *C. haloredivivus* sp. G17. In contrast, only 34% of species (17 of 50) with *LplA* possess *E2*∶10 Sulfolobales, *A. pernix*, 3 *Thermococcus* species, and both *Thermoplasma species*. The absence of *E2* in most Thermococcus species is perhaps explained by the presence of an alternative lipoylation target, the glycine cleavage system protein H (*GcvH*). A comprehensive bioinformatic search for potential lipoyl domains in the genomes of the remaining 20 species without *E2* or *GcvH* revealed a complete absence in 19 of their genomes (the exception being *S. acidocaldarius DSM 639*). This observation is likely explained by a transferase function involving substrates with cryptic lipoylation domains, although it is possible that these represent obsolete lipoylation systems that may be subject to loss through genome reduction mechanisms. Overall, the strong correspondence between E2 and lipoylation systems, particularly in the case of LipM and LipB, suggests a conserved aerobic metabolic functionality of lipoylation systems in these taxa.

### Absence of Lipoylation Systems in Anaerobes

**Figure 3:**
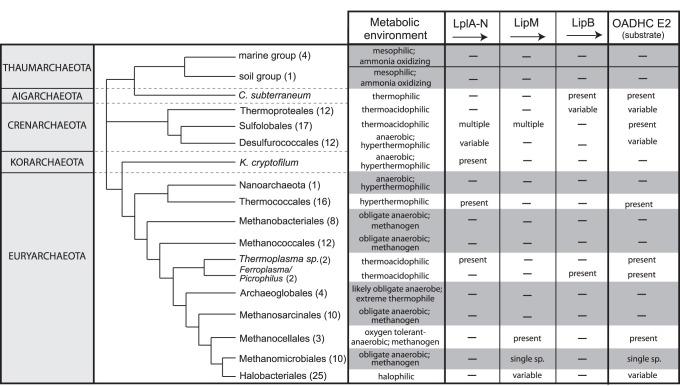
Comparative genomic analysis of lipoylation pathways in archaea. The genomic presence of lipoylation enzymes *LplA-N*, LipM or *LipB* and their substrate OADHC *E2* is indicated. Archaeal orders lacking lipoylation pathways are highlighted (grey shading). The broad metabolic environment of each archaeal order and the number of species analyzed are also indicated. Phylogenetic relationships are based on Brochier-Armanet *et al.* [53]; branch lengths are not drawn to scale.

The absence of a lipoylation pathway in 62 of the 147 species surveyed raised the possibility that loss of lipoylation pathways might be concentrated amongst anaerobes. In support of this assertion, correlated lipoylation pathway absence (including transferase/ligase enzyme and its substrate) was observed in all Thaumarchaeota, Nanoarchaeota, Methanopyrales, Methanobacteriales, Methanococcales, Archaeoglobales, Methanosarcinales, and Methanomicrobiales, all of which are characterized as obligate anaerobes (Fig. 3). Strikingly, amongst methanogens, all sequenced Methanocellales retain *LipM* and *LipA* and have demonstrated oxygen stress tolerance [42], [43], [44]. This raises the possibility that this lipoylation pathway may not be solely associated with energy metabolism *per se*, but rather may be part of a pathway to survive periodic exposure to aerobic conditions. A particularly compelling example that supports selective retention of lipoylation is LipM Clade III where the presence and phylogenetic proximity of LipM in Halobacteriales and Methanocellales is consistent with acquisition of *LipM* prior to the divergence between Methanogen Class II and Halobacteriales, followed by loss in other obligately anaerobic Class II Methanogens (Methanosarcinales and Methanomicrobiales). A similar pattern is observed in LplA Clade I and Clade II (Fig. 2) where LplA sequences from Desulfurococcales, Sulfolobales, and Thermococcales (and *Thermoplasma* in the case of Clade I) cluster monophyletically. This is most parsimoniously explained by presence of *LplA* in the ancestor of Crenarchaeota and Euryarchaeota with subsequent loss in lineages leading to the Methanogens, Archaeoglobales and Halobacteriales (Fig. 3).

### Lipoylation in Aerotolerant Archaea

**Figure 4:**
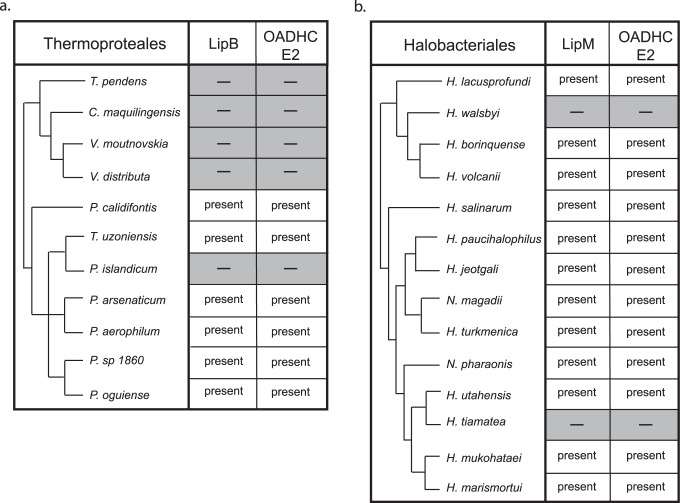
Genomic co-retention of *LipB* and *LipM* lipoylation genes with OADHC *E2*. (A) The presence of the *LipB* and OADHC *E2* genes in sequenced Thermoproteales genomes is indicated (species lacking both are highlighted in grey). *Pyrobaculum sp 1860* and *Pyrobaculum oguniense* have yet to be incorporated into the *Pyrobaculum* phylogeny and have been placed arbitrarily in the *Pyrobaculum* genus. (B) The presence of *LipM* and OADHC *E2* in sequenced Halobacteriales genomes is indicated (species lacking both are highlighted in grey). Phylogenetic relationships are based on Brochier-Armanet *et al.* [53]; branch lengths are not drawn to scale.

Four archaeal orders displayed variable retention of lipoylation systems and, amongst these, there was a strong correspondence between retention of a lipoylation enzyme and its E2 substrate, as would be predicted by their biochemical relationship. In Thermoproteales, all six species retaining *E2* also possess *LipB* (Fig. 4a), with five of these from the *Pyrobaculum* genus. *Pyrobaculum* species are metabolically versatile and grow under both aerobic and anaerobic conditions [45], [46], with the sole exception of *P. islandicum*, a strict anaerobe in which the absence of lipoylation capability is most parsimoniously explained by gene loss [47]. Halobacteriales can generally tolerate aerobic conditions, consistent with the widespread retention of *LipM* and *E2* among Halobacteriales (Fig. 4b). A correlated loss of both *LplM* and *E2* was observed in two Halobacteriales: *Halorhabdus tiamatea*, an anaerobe that inhabits anoxic deep sea brine [48], and *Haloquadratum walsbyi,* which inhabits essentially anoxic environments due to their extremely high salinity [49].

Despite meta-level correspondence between anaerobic metabolism and *E2* lipoylation loss, notable exceptions were identified. As mentioned previously, *LplA* appears to have been retained in 9 of the 10 sequenced anaerobic Desulfurococcale species, despite widespread *E2* loss (Fig. S4). Strikingly, *A. pernix*, the only sequenced strictly aerobic Desulfurococcale [50], retains *LplA* and the *OADHC* operon. *LplA* has been retained in all sequenced species of Thermococcales but only three possess *E2*, a finding consistent with anaerobic conditions for most Thermococcales (Table S1). Unlike the better studied Crenarchaeota and Euryarchaeota, restricted genomic and environmental data exist for the more recently identified Korarchaeota, Aigarchaeota and Thaumarchaeota [51,52,53 and reviewed by 54]. Consistent with a general correspondence between E2 lipoylation and aerobic metabolism, analysis of the strictly anaerobic *Korarchaeum cryptofilum* [51], the only Korarchaeota species with an available genome, revealed an absence of lipoylation and *OADHC* genes. *Caldiarchaeum subterraneum*, the only representative of the proposed phylum Aigarchaeota, has features distinguishing it from the Thaumarchaeota, such as a ubiquitin-like protein modifier system [52] and genes encoding LipB and OADHC components. Previous analysis of the *Caldiarchaeum subterraneum* genome suggested versatile energy metabolism [52] including an almost complete Emden-Meyerhof pathway and a complete citric acid cycle, and our identification of an E2 lipoylation pathway is consistent with aerobic metabolism. In contrast to these Korarchaeota and Aigarchaeota examples, Thaumarchaeota species lack lipoylation and *OADHC* genes despite the fact that they inhabit diverse environments, ranging from aerated soils to oxygen-depleted marine sediment [54]. This observation is explained by their ability to oxidize ammonia (and potentially related substrates) and the adaptation of Thaumarchaeota ecotypes to diverse abiotic conditions, including low ammonia and low oxygen environments [55], [56]. As such, this largely autotrophic basal archaeal clade has the unique biological ability to oxidize reduced nitrogen species and presumably has no evolutionary reliance on aerobic metabolic pathways associated with OADHC complexes.

### Genomic Heterogeneity of the LplA Lipoylation System

In contrast to widespread reductions of genome complexity in archaea, including E2 lipoylation loss across diverse anaerobes, our analyses also revealed substantial *LplA* copy expansion and heterogeneity across a restricted set of species possessing this gene. As a likely result of gene duplication events, *LplA* and *LipM* copy number varies across Sulfolobales and *A. pernix*. This variation may ultimately prove to be an adaptive response relating to the availability of exogenous lipoic acid and endogenous octanoic acid. Our analysis also confirmed the previous identification of a single Sulfolobale *LplA* copy encoding both N- and C-terminal domains of LplA and identified similar genes in a Thermococcale (*gammatolerans EJ3*), a Halobacteriale (two copies in *turkmenica DSM5511*) and a Desulfurococcale (*A. pernix*). Previous phylogenetic analyses have supported the proposition that a bipartite gene system, comprising *LplA-N* and *LplA-C*, predates the origin of the *LplA* gene found in most bacteria and eukaryotes that encodes both LplA domains [23]. The widespread presence of the bipartite *LplA-N/C* system in archaea, and evidence presented supporting its ancient evolutionary origins,are consistent with this scenario and are further supported by our identification of numerous bacterial species, such as *Bordetella*, *Achromobacter* and *Rhodanobacter*, which also possess a bipartite *LplA* gene system (data not shown). It is therefore possible that distinct genes encoding the N- and C-terminal domains have formed chimeric proteins in several archaeal lineages. Relevant to the possibility of chimeric fusions is the observation that genomic rearrangements have resulted in co-localization of *LplA-N* and *LplA-C* multiple times during archaeal evolution. Transcriptional coupling of *LplA-N* to *LplA-C* is present in *T. acidophilum* (supported by out-of-frame coding sequence overlap (1 base pair), a readily identifiable TATA box upstream of the *LplA-C* gene, and the absence of identifiable *cis*-regulatory sequences proximal to the 5′ end of *LplA-N* [24]) and our analysis revealed an independent origination of transcriptional coupling of *LplA-N* to *LplA-C* genes in five Desulfurococcales. The monophyletic relationship amongst these species is consistent with the co-localization of these genes in their common ancestor (Fig. S4). Therefore, *LplA* and *LipM* gene duplications appear to occur in a restricted set of taxa and targeted experiments will be necessary to assess a possible association of this with differential oxygen tolerance capacities amongst these species.

## Discussion

Metagenomic sequencing of microbial communities has progressed beyond the initial goals of assessing species composition to the more penetrating proposition that biotic and abiotic interactions can be modelled based on metagenomic data. Given the overwhelming complexity of such ecological and environmental interactions, the accuracy of such inferences needs to be initially assessed using relatively straightforward interactions, mediated by well studied pathways, in a set of organisms likely to exhibit marked diversity in the relevant interactions. We have therefore investigated aerobiosis capacity across a diverse set of archaeal genomes using the well-characterized enzymes responsible for OADHC lipoylation. The potential of using metagenomic data to establish links between metabolic capabilities and environmental conditions is of particular importance to archaea, which are often difficult to culture in the laboratory and therefore remain refractory to direct analysis [57].

OADHC lipoylation is essential for metabolism in aerobic bacteria and eukarya, making it a compelling candidate system to assess the potential for more expansive metagenomic analyses across the archaea. Our analysis revealed three broad trends, which together suggest that metagenomic inferences have the potential to be informative when well understood pathways are interrogated in organisms possessing relevant environmental/ecological diversity. First, the retention of a single lipoylation pathway (*LplA*, *LipB* or *LipM*) in species capable of lipoylation is consistent with genome streamlining during archaeal evolution. As such, the presence of genes, pathways or networks within archaeal genomes (and the concomitant absence of redundancy) can be generally attributed to the selective retention of essential functions. Second, the rather widespread presence of a *LipM/LipB-LipA* system provides support for the presence of FA biosynthesis and endogenous octanoic acid across a surprisingly diverse range of archaea. Third, OADHC E2 lipoylation has been consistently lost in obligate anaerobes and may therefore serve as a diagnostic metagenomic marker for aerobiosis. Similarly, the presence of the lipoylation/OADHC system in organisms previously thought to be strict anaerobes may indicate the existence of mechanisms for oxygen tolerance, but may also reflect previously unrecognized aerobic respiration capabilities. In addition to our observation, aerotolerance has been attributed to superoxide reductase in some species of Methanosarcinales, indicating that distinct mechanisms leading to oxygen stress adaptation may exist [58]. As metagenomic approaches often result in fragmented genome sequences, inferring gene or pathway absence may be difficult, making arguments based on gene presence (in this case indicating aerobiosis or oxygen tolerance) more reliable. It should be noted that our analysis relied largely upon complete genomes, although uncertainty associated with the analysis of incomplete sequences may still apply for species derived from ecological samples. In conclusion, given highly variable retention of gene repertoires across the archaea, extension of comparative genomic approaches to broader metabolic and homeostasis networks should be useful in revealing genome-wide characteristics related to archaeal adaptation to diverse environments.

Our analysis demonstrates that the evolution of archaeal lipoylation systems is generally in agreement with major trends identified in recent reconstructions of archaeal genome evolution [9]. An increase in genomic complexity (the innovation phase) is evidenced by multiple lipoylation system acquisitions that have involved all the primary lipoyl-octanoyl transferase systems (*LplA*, *LipM* and *LipB*). It is noteworthy that, based on our phylogenetic analyses, these systems are inferred to be largely eubacterial in origin and appear to have been acquired from a diverse range of bacterial phyla, including Firmicutes, Actinobacteria and Proteobacteria. This period of increased complexity in lipoylation genetics was then followed by a reductive phase where lipoylation systems were lost across a diverse range of archaeal species, most notably those that have become adapted to an obligately anaerobic life history. Gene loss may therefore have played a prominent role in the functional diversification of archaea during their adaptation to, and exploitation of, diverse and often extreme habitats.

## Supporting Information

Figure S1 **LplA-N protein alignment.** LplA-N sequences were included from one representative species for each of the six archaeal orders in Fig. 1 that retain LplA (*P. horikoshii*, *Pyrococcus horikoshii*, a Sulfolobale; *M. arvoryzae*, *Methanocella arvoryzae*, a Methanocellale; *H. butylicus*, *Hyperthermus butylicus*, a Desulfurococcale; *S. solfataricus*, *Sulfolobus solfataricus*, a Sulfolobale; *T. acidophilum*, *Thermoplasma acidophilum*, a Thermoplasmatale; *N. pharaonis*, *Natronomonas pharaonis*, a Halobacteriale). Conserved amino acid residues are highlighted. Secondary structure elements (α-helices α1 to α8, β-strands β1 to β10, and 310-helices η1 to η3) from T. acidophilum LplA-N in the structure of the *T. acidophilum* LplA-N:LplA-C complex (PDB code 3R07) are shown.
(EPS)

Figure S2 **Maximum Likelihood Phylogeny of LplA and LipM.** Phylogenetic tree including 132 LipM and LplA sequences from archaea, LplA sequences from major eukaryotic species (*S.cerevisiae*, *D. melanogaster*, *M. musculus* and *H. sapiens*) and LplA and LipM sequences from eubacteria representing actinobacteria (*S. coelicolor*), bacteroidetes (*B. thetaiotaomicron*), firmicutes (*B. subtilis* and *S. aureus*) and proteobacteria (*E. coli* and *B. pseudomallei*). Bootstrap values are provided and species abbreviations can be found in Supplemental Table S1.
(EPS)

Figure S3 **Maximum Likelihood Phylogeny of LipB.** Phylogenetic tree including 13 LipB sequences from archaea and LipB sequences from major eukaryotic species (*S.cerevisiae*, *D. melanogaster*, *M. musculus* and *H. sapiens*) and eubacteria representing actinobacteria (*S. coelicolor*), bacteroidetes (*B. thetaiotaomicron*) and proteobacteria (*E. coli* and *B. pseudomallei*). Putative horizontal gene transfer events are indicated (asterisks). Bootstrap values are provided and species abbreviations can be found in Supplemental Table S1.
(EPS)

Figure S4 **Desulfurococcales retention of LplA and OADHC operons.** The ancestral lineage of the five monophyletic species (red cross) where LplA-N and LplA-C are inferred to have become co-localized and transcriptionally coupled.
(EPS)

Table S1 **Lipoylation systems and substrates in Archaea.** A complete inventory of the species analyzed, the lipoylation related proteins identified and evidence supporting horizontal gene transfer.
(XLS)
