# Correction: Comparative Genomic Analysis Reveals 2-Oxoacid Dehydrogenase Complex Lipoylation Correlation with Aerobiosis in Archaea

**DOI:** 10.1371/journal.pone.0100680

**Published:** 2014-06-13

**Authors:** The PLOS ONE Staff

There are errors in Figure 2. The authors have provided a corrected version here.

**Figure 2:**
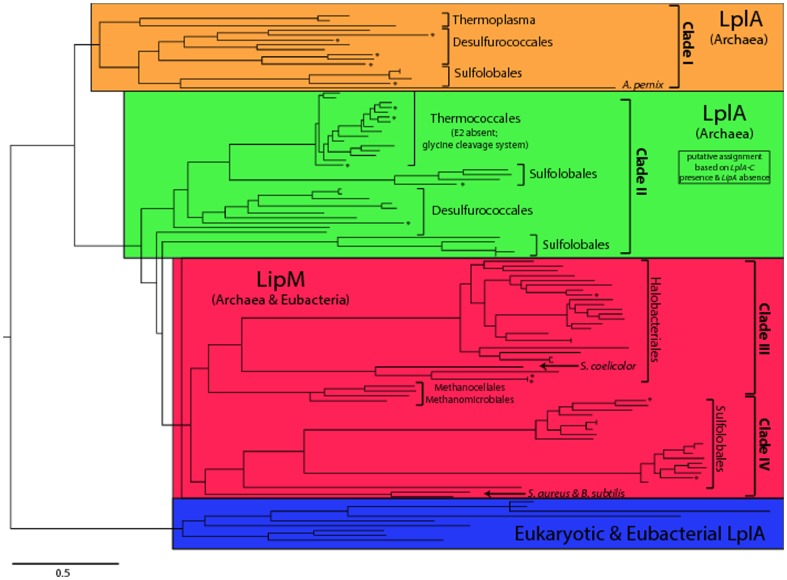
Phylogenetic analysis of LipM and LplA. Maximum likelihood phylogenetic tree including 131 LipM and LplA sequences from archaea, LplA sequences from major eukaryotic species (*S. cerevisiae, D. melanogaster*, *M. musculus* and *H. sapiens*) and LplA and LipM sequences from eubacteria representing Actinobacteria (*S. coelicolor),* Bacteroidetes (*B. thetaiotaomicron*), Firmicutes (*B. subtilis* and *S. aureus*) and Proteobacteria (*E. coli* and*B. pseudomallei).* Putative cases of horizontal gene transfer are indicated (asterisk) and major phylogenetic clades are highlighted: archaeal LplA (Clade I - orange; Clade II - green), LipM (red), and eukaryotic and eubacterial LplA (blue). The full phylogenetic tree including species names and bootstrap values is provided Figure S2.
